# 

**Published:** 1893-12

**Authors:** 


### CONTENTS.

*EDITORIAL.*

| | PAGE |
|---|---|
| Address to Delinquent Subscribers, . . . . . . . . | 561 |
| Wells on Intermittent Fever, . . . . . . . . . . | 562 |

*MISCELLANEOUS.*

| | |
|---|---|
| Introductory to the 46th Annual Course of Instruction in the Hahnemann Medical College of Philadelphia. B. Frank Betts, M. D., | 563 |
| Potentiation Physiologically Proven. Prof. Dr. Gustav Jaeger. Translated by B. Fincke, M. D., . . . . . . . | 577 |
| Nosode Prescribing. Proceedings I. H. A., . . . . . . | 580 |
| In Memoriam—Samuel Swan, M. D. Harlyn Hitchcock, M. D., . | 589 |
| New York Homœopathic Union, . . . . . . . . | 594 |
| Value of Vaccination, . . . . . . . . . . . | 598 |
| Resolutions of New York State Society. John L. Moffat, M. D., . | 599 |

*BOOK NOTICES.*

| | |
|---|---|
| Essentials of Minor Surgery. By Edward Martin, A. M., M. D., . | 599 |
| Therapeutics of Cholera. By P. C. Majumdár, M. D., . . . . | 600 |

*NOTES AND NOTICES.*

| | |
|---|---|
| Mr. W. B. Saunders—Dr. Arthur G. Allan—Reed & Carnrick's Infant Food—Mr. W. B. Saunders, . . . . . . . . | 600 |

